# Parallel Loop Binding Compression Suture, a Modified Procedure for Pernicious Placenta Previa Complicated With Placenta Increta

**DOI:** 10.3389/fsurg.2021.786497

**Published:** 2021-11-29

**Authors:** Mengdi Fu, Hualei Bu, Yan Fang, Chunling Wang, Li Zhang, Yang Zhang, Xiao Sun, Mingbao Li, Chengjuan Jin, Yintao Xu, Lijun Chen

**Affiliations:** ^1^Department of Gynecology and Obstetrics, Qilu Hospital, Cheeloo College of Medicine, Shandong University, Jinan, China; ^2^Department of Anesthesia, Qilu Hospital, Shandong University, Jinan, China; ^3^Department of Radiology, Qilu Hospital, Shandong University, Jinan, China; ^4^Department of Ultrasound, Qilu Hospital, Shandong University, Jinan, China; ^5^Department of Obstetrics and Gynecology, Shanghai General Hospital, School of Medicine, Shanghai Jiao Tong University, Shanghai, China

**Keywords:** parallel loop binding compression suture, pernicious placenta previa, placenta increta, cesarean section, newborns

## Abstract

**Objective:** To evaluate the efficacy and safety of parallel loop binding compression suture of the lower uterus during cesarean section in pernicious placenta previa complicated with placenta increta.

**Methods:** This retrospective study was performed in patients with pernicious placenta previa complicated with placenta increta or percreta between November 2014 and December 2020 at the Qilu Hospital of Shandong University. Patients underwent parallel loop binding compression suture surgery were defined as study group, and patients underwent traditional surgery with figure-of-eight sutures as the main hemostatic method were defined as control group. Postpartum hemorrhage was evaluated as the primary outcome. The secondary outcomes included age, gestational weeks, operative time, fetal childbirth time, prevention of hysterectomy, blood transfusion, duration of postoperative catheterization, duration of antibiotic treatment, and postoperative hospitalization (days). Additionally, neonatal outcomes were evaluated.

**Results:** A total of 124 patients were enrolled in the study, including 38 patients receiving parallel loop binding compression suture surgery in the study group, and 86 patients in the control group. With parallel loop binding compression suture, the average operation time was significantly reduced (109.0 ± 33.5 vs. 134.4 ± 54.2 min, *p* = 0.00), and the volume of blood lost were also decreased (2152.6 ± 1169.4 vs. 2960.5 ± 1963.6 ml, *p* = 0.02), which correspondingly reduced RBC transfusion (7.2 ± 3.5 vs. 10.3 ± 8.7 units, *p* = 0.03) and FFP transfusion (552.6 ± 350.3 vs. 968.0 ± 799.8 ml, *p* = 0.00). The fetal childbirth time was extended (14.1 ± 5.6 vs. 11.0 ± 8.0 min, *p* = 0.03), however, there was no increase in NICU admission rates (36.9 vs. 34.9%, *p* = 0.83). Except for one premature infant (32 weeks) death in the control group, all infants at our hospital were safely discharged after treatment.

**Conclusion:** Parallel loop binding compression suture is an effective, swift, practical, and safe method to reduce postpartum bleeding in women with pernicious placenta previa, complicated with placenta increta. Besides, it has no adverse effects on newborns.

## Introduction

Placenta previa is an obstetric complication in which the placenta is partially or wholly inserted in the lower uterine segment (1, 2). In the past decades, women have increasingly opted for cesarean section (CS), leading to an increasing incidence of placenta previa (3, 4). Globally, postpartum hemorrhage accounts for the death of 140,000 women annually (5). Placenta previa is an independent risk factor for maternal hemorrhagic morbidity (6) and can also lead to morbidity and mortality in pregnant women and neonates (7).

Pernicious placenta previa is a special type of placenta previa, in which the placenta attaches to previous cesarean delivery scars (8), remaining a challenge in obstetric practice. Placenta increta, whereby the villi invade the myometrium, is a type of accreta placentation (9). Pernicious placenta previa complicated with placenta accreta, particularly placenta increta or percreta, has a high risk of maternal hemorrhage prior to, during, and after CS (10).

In previous decades, various surgical methods have been proposed to control bleeding associated with placenta previa, including hysterectomy (11–13). Nevertheless, during the operative treatment of pernicious placenta previa, reducing blood loss remains a challenge. However, there is also the strong desire to preserve the uterus and fertility; therefore, an alternative to hysterectomy is required. This study aimed to evaluate the effectiveness of parallel loop binding compression suture of lower uterus regarding control of bleeding and prevention of hysterectomy during cesarean section in pregnant women with pernicious placenta previa, complicated with placenta increta.

## Patients and Methods

### Patients

**Figure 1:**
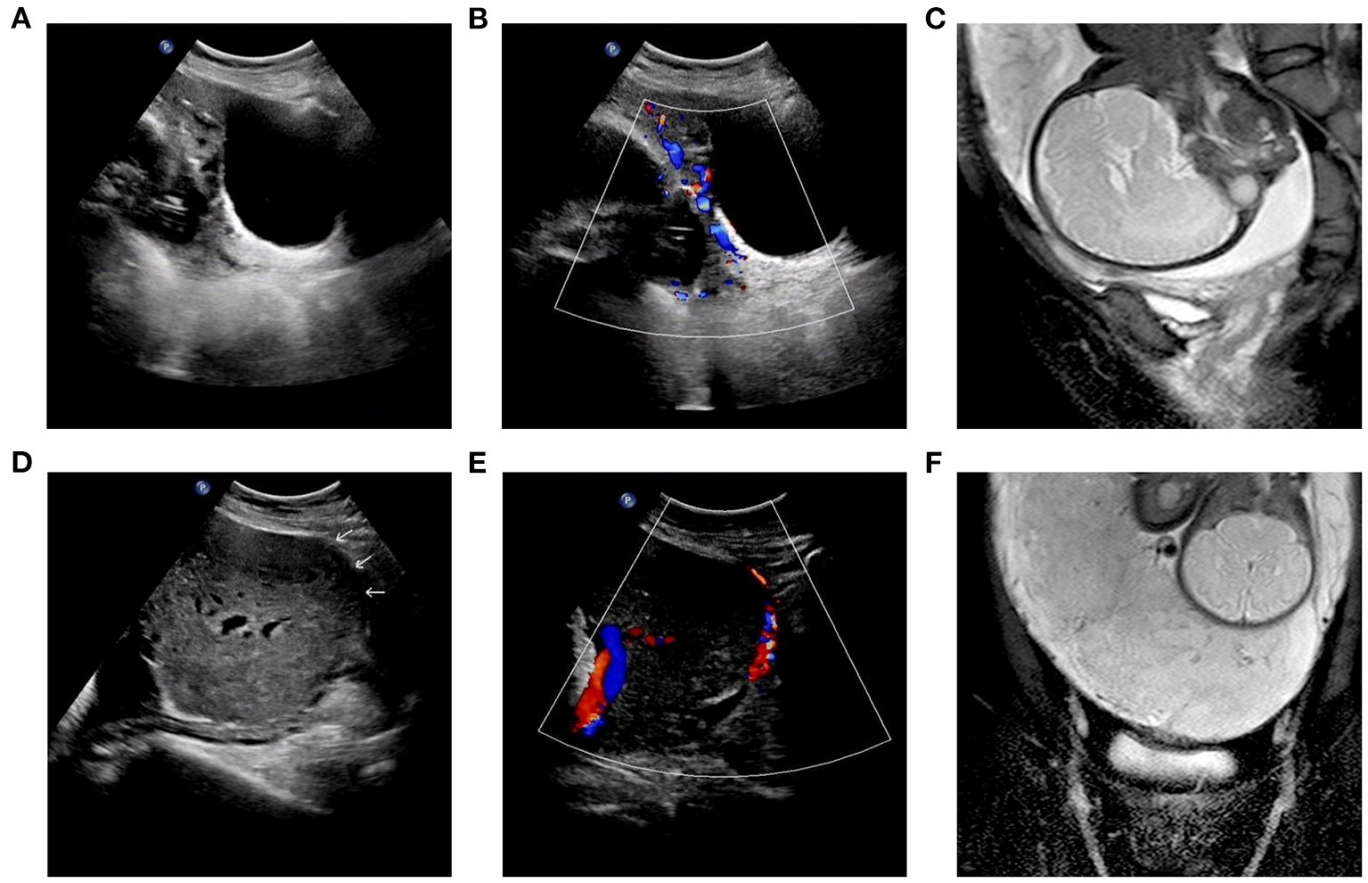
**(A–C)** Patient No.16 of study group: marginal placenta previa. The placenta invaded the serous layer of the uterus and the posterior wall of the bladder, and the adjacent areas showed rich blood flow signals. **(D–F)** Patient No.23 of study group: complete placenta previa. The placenta invaded the serous layer of the uterus, and the adjacent areas showed rich blood flow signals.

**Figure 2:**
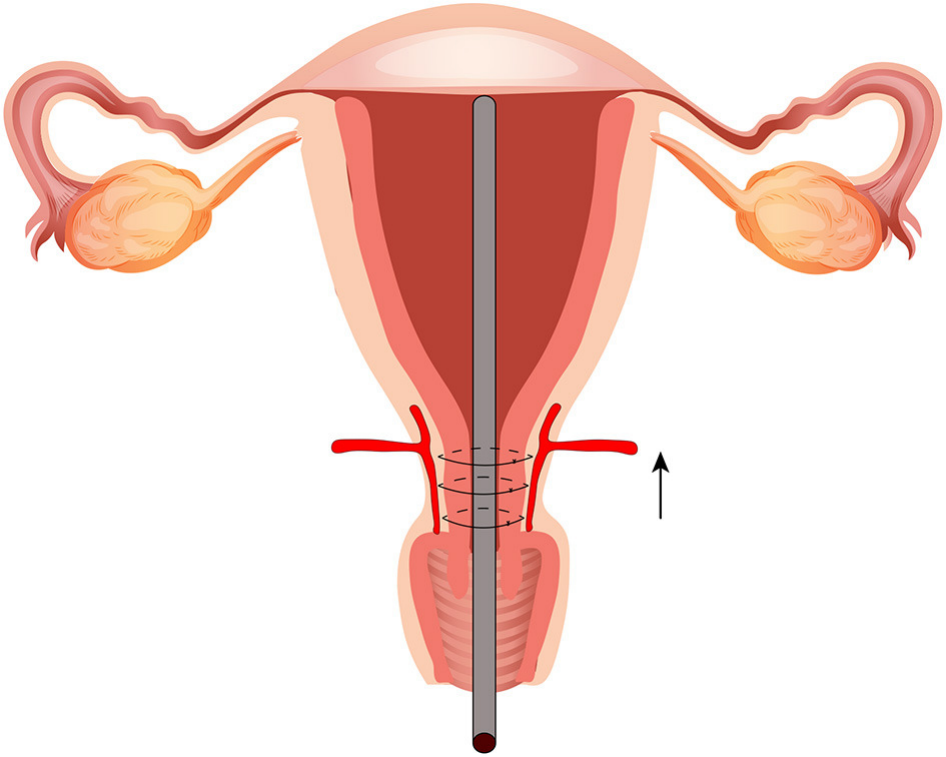
Parallel loop binding compression suture procedure.

Between November 2014 and December 2020, patients at Qilu Hospital of Shandong University fulfilled with the following criteria were enrolled in this study: (1) diagnosis of pernicious placenta previa by ultrasound and/or magnetic resonance imaging (MRI), the specific image performance is shown in Figure 1; (2) placenta increta or placenta percreta complication. Emergency surgeries were excluded from the study. Patients underwent parallel loop binding compression suture surgery were defined as study group, and patients underwent traditional surgery with figure-of-eight sutures as the main hemostatic method were defined as control group. The application of this suturing technique was approved by the hospital's ethics committee, and all patients provided written informed consent before surgery.

### Data Collection

The following clinical characteristics of patients and newborns were routinely collected, including age, history of gestation, number of cesarean sections, time since last CS (years), weeks of gestation, use of balloon occlusion of the abdominal aorta, operation time (min), adjunctive hemostatic procedures, blood transfusion (units), estimated blood loss (mL), duration of indwelling urethral catheter (days), postoperative hospital stay (days), duration of antibiotic use (days), neonatal birth weight, Apgar scores at 1- and 5 min, and duration of hospitalization (days) at the neonatal intensive care unit (NICU).

Normally distributed data are presented as means ± standard deviation and medians and interquartile ranges (IQRs), whereas categorical variables are expressed as numbers and/or percentages. Statistical analyses were performed using SPSS 26.0 (IBM SPSS Statistics, Armonk, NY, USA). Significance levels were ^*^p < 0.05; ^**^p < 0.01.

### Parallel Loop Binding Compression Suture Procedure

An experienced multidisciplinary team involving anesthetists, interventional radiologists, hematologists, obstetricians, gynecologists, and neonatologists was consulted to prepare for the surgery. The decision whether to preset the abdominal aorta balloon was made by senior obstetrics professionals based on imaging and clinical characteristics of the patient. Balloon catheter occlusion of the abdominal aorta was performed by an interventional radiologist on the day of surgery. Elective cesarean section was preferentially routinely performed during weeks 35–38, but it also depended on the patient's clinical symptoms.

**Figure 3:**
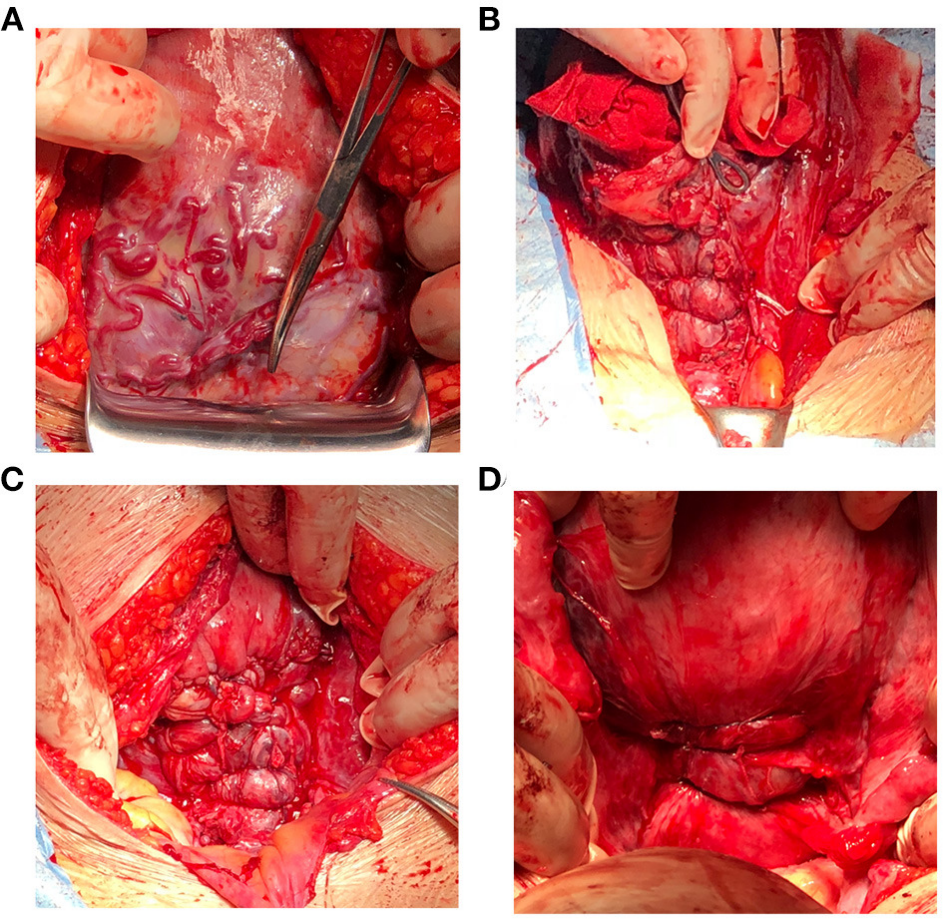
Corresponding surgical pictures. **(A)** Pernicious placenta previa complicated with placenta increta. **(B,C)** The anterior wall of uterus after parallel loop binding compression suture was performed, and adequate hemostasis effect was achieved. **(D)** The posterior wall of uterus after parallel loop binding compression suture was performed.

To expose the lower part of the uterus, the bladder is pushed down through top or side approach; the broken blood vessels on the bladder are ligated simultaneously, and the bladder is pushed down to the maximum extent. The exposure operation is aborted if the lower segment of the uterus ruptures or bleeding becomes difficult to control. The abdominal aorta balloon is inflated (if preset before surgery). When incising the uterus, the placenta should be avoided. If it is difficult to avoid the placenta, the placenta should be punched, and the fetus delivered quickly. The uterus is quickly moved out of the abdominal cavity and the lower segment of the uterus is bound with occluding cuff. The placenta is quickly removed, and the adhesion or implantation region cleaned. The bladder is pushed down until it is below the placenta attachment site. A Fr28 abdominal drainage silicone tube is placed from the uterine cavity to the vagina and a parallel loop binding suture ligation applied from the cervix to the lower part of the uterus. The first needle is inserted laterally near the front of the lower margin of the cervix, walked along the lateral wall, unto the lateral posterior of the cervix. The same procedure is performed ~1 cm above the first suture site and is repeated 3–4 times to completely seal the bleeding on the placental dissection surface of the upper cervix and lower uterus, and then the weak myometrium is sutured and reinforced. Finally, the ascending branches of the bilateral uterine arteries are sutured (if necessary). B-Lynch uterine suture is performed (if necessary). Uterine cavity drains are kept for 4–6 h, and the Fr28 abdominal drainage silicone tube is subsequently removed (see Figures 2, 3 and Supplementary Videos 1–3).

### Follow-Ups

The first follow-up for all women was conducted at 6 weeks after the cesarean section and ultrasound was performed. Additional follow-up was conducted every 3 months for the first year and annually by telephone until May 2021.

## Results

### Baseline Characteristics Between Two Study Groups

A total of 124 patients fulfilled with the criteria were included in the study, 38 of whom underwent parallel loop binding suture surgery, and another 86 patients underwent traditional surgery with figure-of-eight sutures as the main hemostatic method. The comparisons of surgical information between two study groups were listed in Table 1, and the detailed characteristics of the study group and control group were shown in Table 2 and Supplementary Material 1, respectively.

**Table 1:** Comparisons of surgical information between study and control groups.

**Characteristic**	**Study group** (***N*** **= 38**)	**Control group** (***N*** **= 86**)	***P***
Age (years)	34.6 ± 4.4	34.0 ± 4.6	0.53
GA (weeks)	36.3 ± 1.7	36.3 ± 1.4	0.81
No. of CS	1.3 ± 0.5	1.4 ± 0.6	0.66
Antibiotic (days)	4.7 ± 1.6	4.7 ± 1.4	0.95
Post-operation hospitalization (days)	6.6 ± 2.7	6.5 ± 2.7	0.74
Neonatal birth weight (g)	3,000.9 ± 530.2	2,948.3 ± 469.8	0.58
Fetal childbirth time (min)	14.1 ± 5.6	11.0 ± 8.0	0.03^*^
Total time of operation (min)	109.0 ± 33.5	134.4 ± 54.2	0.00**
Urethral catheter (days)	2.5 ± 1.2	4.1 ± 2.7	0.00^**^
Blood loss (ml)	2,152.6 ± 1,169.4	2,960.5 ± 1,963.6	0.02^*^
RBC Transfusion (unit)	7.2 ± 3.5	10.3 ± 8.7	0.03^*^
FFP Transfusion (ml)	552.6 ± 350.3	968.0 ± 799.8	0.00^**^

*GA, Gestational age; CS, Cesarean section; RBC, Red blood cell; FFP, Fresh frozen plasma*.

* *p <0.05;*

** *p <0.01*.

**Table 2:** Clinical characteristics of study group.

**No**.	**Age**	**GA (week)**	**G/P/A**	**No. of CS**	**Fetal childbirth time(min)**	**Total time (min)**	**Blood loss (ml)**	**Blood transfusion** **(unit PRBC/ml FFP)**	**Urethral catheter** **(days)**	**Antibiotic** **(days)**	**Post-operation** **(days)**
1	28	36 + 2	2/1/0	1	12	83	1,000	2/0	2	4	4
2^a^	35	36 + 6	4/2/1	2	13	115	2,000	8/800	2	6	8
3	38	35 + 2	7/2/4	2	14	85	2,600	8/800	3	3	5
4	35	33 + 4	5/2/2	2	15	100	1,000	6/750	3	4	5
5	34	35 + 4	6/2/3	2	12	72	800	4/600	1	3	3
6	33	35 + 4	3/1/1	1	13	95	2,000	8/400	2	3	5
7	34	37 + 3	3/2/0	1	11	92	1,800	4/400	3	5	5
8^a^	35	32 + 6	5/3/1	2	15	105	2,000	8/800	4	8	8
9^a^	45	36 + 5	3/2/0	1	5	70	1,500	8/800	3	7	10
10	36	37 + 3	3/2/0	2	8	138	3,000	12/1,000	3	4	5
11	36	37 + 4	4/1/2	1	11	85	2,000	6/800	1	4	4
12	44	35 + 6	4/2/1	2	22	161	3,000	12/1,000	4	6	8
13	41	32 + 6	4/2/1	1	5	67	1,200	4/400	3	4	8
14	29	35 + 2	3/2/0	2	23	110	1,500	6/400	2	3	5
15	41	33 + 2	6/2/3	2	13	105	1,500	6/400	1	7	17
16	34	37 + 3	3/1/1	1	10	115	3,000	10/400	2	4	5
17^a^	35	37 + 4	5/2/2	2	10	105	2,000	6/400	7	8	8
18^a^	26	39 + 4	2/1/0	1	7	74	500	4/0	1	3	7
19^a^	28	39 + 6	4/1/2	1	12	107	2,000	8/800	4	4	4
20^a^	38	36 + 2	2/1/0	1	11	115	1,800	4/400	2	4	4
21^a^	32	36	3/2/0	2	10	113	1,800	8/800	3	5	5
22^a^	27	37 + 2	3/1/1	1	12	127	1,000	4/400	4	3	4
23^a^	29	36 + 5	2/1/0	1	12	102	700	4/0	2	7	12
24^b^	34	34 + 3	3/1/1	1	30	195	6,000	14/1,400	2	5	8
25	35	37 + 6	3/1/1	1	18	75	800	4/0	3	3	3
26	35	34 + 5	3/1/1	1	11	105	2,000	4/0	1	3	7
27	33	33	5/1/3	1	18	90	2,000	4/400	2	4	7
28	32	35 + 5	3/1/1	1	20	95	1,500	4/400	2	3	7
29	35	37 + 1	3/1/1	1	16	105	1,500	4/400	2	4	7
30	39	37 + 2	4/1/2	1	20	105	3,000	10/1,050	3	4	7
31^b^	37	35 + 6	3/1/1	1	15	150	5,000	16/1,200	2	5	7
32^b^	36	38 + 4	5/1/3	1	27	225	4,000	14/800	3	3	7
33^c^	37	35 + 4	6/2/3	2	13	144	3,000	12/800	1	5	7
34	29	37	3/1/1	1	13	70	1,800	4/400	2	5	7
35	33	37 + 4	5/1/3	1	8	85	2,000	6/200	3	4	5
36	31	38 + 1	2/1/0	1	10	118	2,500	6/400	2	5	5
37	35	37	4/2/1	2	22	97	3,000	8/400	2	6	9
38^d^	39	37 + 2	4/1/2	1	17	145	4,000	12/1,000	2	9	10

a *Abdominal aortic balloon was placed preoperatively*.

b *Hysterectomy was performed due to massive blood loss*.

c *20 units of cryoprecipitate were infused*.

d *20 units of cryoprecipitate and 1 unit of platelet were infused*.

*G/P/A, Gravidity/Parity/Abortion; CS, Cesarean section*.

The mean age of the study group and the control group were 34.6 (SD: ±4.4, range: 26–45) and 34.0 (SD: ±4.6, range: 22–46) years, respectively, with no statistical difference (*p* = 0.35), and the gestational weeks of termination were relatively consistent (36.3 ± 1.7 vs. 36.3 ± 1.4, *p* = 0.81). There were 13 cases (34.2%) in the study group and 26 cases (30.2%) in the control group of patients with more than one cesarean section, without statistical difference between the two groups (*p* = 0.81). Based on the imaging and clinical characteristics of the patients, an abdominal aortic balloon was placed preoperatively in 10 women (26.3%) of study group, and 15 women (17.4%) of control group (*p* = 0.26).

### The Advantages of Parallel Loop Binding Compression Suture

The average operation time was 109.0 ± 33.5 min with parallel loop binding compression suture, which was significantly reduced compared with traditional operation method (134.4 ± 54.2 min, *p* = 0.00). The volume of blood lost were also decreased significantly (2,152.6 ± 1,169.4 vs. 2,960.5 ± 1,963.6 ml, *p* = 0.02), which correspondingly reduced RBC transfusion (7.2 ± 3.5 vs. 10.3 ± 8.7 units, *p* = 0.03) and FFP transfusion (552.6 ± 350.3 vs. 968.0 ± 799.8 ml, *p* = 0.00). Besides, parallel loop binding compression suture could shorten the duration of postoperative catheter indwelling (2.5 ± 1.2 vs. 4.1 ± 2.7 days, *p* = 0.00). Due to the hospital's routine surgical procedures, the durations of postoperative antibiotic use (4.7 ± 1.6 vs. 4.7 ± 1.4 days, *p* = 0.95) and postoperative hospitalization (6.6 ± 2.7 vs. 6.5 ± 2.7 days, *p* = 0.74) were consistent both in the study group and control group.

In the study group and the control group, 3 (7.9%) and 6 (7.0%) patients underwent hysterectomy due to massive blood loss, respectively, with similar proportions (*p* = 0.86), suggesting that parallel loop binding compression suture is also effective in avoiding hysterectomy compared with traditional surgery methods.

The most recent postoperative follow-up of study group was updated in May 2021, and the postoperative menstrual cycle and menstrual flow of patients both returned to normal levels.

### Parallel Loop Binding Compression Suture had No Adverse Effect on Neonatal Prognosis

The corresponding dominant characteristics of the newborns were shown in Table 3 and Supplementary Material 2. Because the bladder should be pushed down to the maximum extent to expose the lower part of the uterus in parallel loop binding compression suture procedure, the fetal childbirth time was extended (14.1 ± 5.6 vs. 11.0 ± 8.0 min, *p* = 0.03), however, there was no increase in NICU admission rates (36.9 vs. 34.9%, *p* = 0.83). Multiple neonatal complications were common in preterm infants, which delayed the discharge time. Except for one premature infant (32 weeks) death in the control group, all infants at our hospital were safely discharged after treatment.

**Table 3:** Neonatal characteristics of study group.

**No**.	**Neonatal birth weight(g)**	**Apgar scores (1 min/5min)**	**Neonatal complication**	**hospitalization days in NICU**	**Prognosis**
1	2,900	10/10	None	0	Cure
2	2,270	6/8	1;2	21	Cure
3	2,690	5/8	3	12	Cure
4	2,100	8/10	4	12	Condition improved
5	4,000	9/10	3	0	Cure
6	2,900	10/10	2	7	Cure
7	3,400	10/10	None	0	Cure
8	2,150	7/9	5	17	Condition improved
9	3,400	10/10	None	0	Cure
10	3,000	10/10	None	0	Cure
11	3,400	10/10	None	0	Cure
12	2,700	6/8	1;2;4;5	36	Cure
13	1,900	8/8	None	Unknown	Unknown
14	3,000	10/10	6	8	Cure
15	2,625	10/10	3	13	Cure
16	3,900	8/9	None	0	Cure
17	3,100	10/10	None	0	Cure
18	3,200	10/10	None	0	Cure
19	3,500	10/10	None	0	Cure
20	3,200	10/10	None	0	Cure
21	2,550	9/10	None	0	Cure
22	2,500	10/10	None	8	Cure
23	3,400	10/10	None	0	Cure
24	2,500	4/5	3;7	8	Cure
25	3,300	9/10	None	0	Cure
26	2,650	7/9	3	10	Cure
27	2,200	7/8	3;8;9	14	Cure
28	2,800	10/10	None	0	Cure
29	2,900	10/10	None	0	Cure
30	3,050	10/10	None	0	Cure
31	3,000	10/10	None	0	Cure
32	3,900	10/10	None	0	Cure
33	2,800	10/10	2;3	10	Cure
34	3,900	10/10	None	0	Cure
35	3,200	10/10	None	0	Cure
36	3,050	10/10	None	0	Cure
37	3,800	10/10	None	0	Cure
38	3,200	10/10	None	0	Cure

*Neonatal complication: 1, Neonatal hypoxic-ischemic encephalopathy; 2, Neonatal hyperbilirubinemia; 3, Neonatal pneumonia; 4, Neonatal anemia; 5, Neonatal feeding intolerance; 6, Neonatal diarrhea; 7, Neonatal toxic erythema; 8, Neonatal respiratory distress syndrome; 9, Neonatal apnea*.

## Discussion

Pernicious placenta previa remains a life-threatening obstetric problem, particularly in women with placenta increta. The increasing application of CS and medical abortion had a direct effect on the incidence of all grades of placenta accrete (14). Placental abnormality, including placenta accreta and placenta previa, is one of the most important causes of postpartum hemorrhage (15). Severe bleeding may lead to diffuse intravascular coagulation, multiple organ failure, and even death (16). Massive transfusion and aggressive surgical management, such as hysterectomy, have been the primary treatment to manage severe postpartum bleeding. Up to now, it is the first study to evaluate the safety and effectiveness of parallel loop binding compression suture in patients with pernicious placenta previa, complicated with placenta increta, which was proved to be effective, practical, and safe.

Various surgical sutures have been developed to reduce postpartum hemorrhage and preserve fertility in patients with placental abnormality during CS. Cho et al. (17) performed hemostatic multiple square suturing in women with postpartum hemorrhage who did not respond to conservative management during CS. However, the study was based on experiences from a limited number of women with placenta previa. Therefore, it could not be concluded that multiple square suturing can effectively treat postpartum hemorrhage in placenta previa. Hwu et al. (18) reported that parallel vertical compression sutures were used to control postpartum hemorrhage in women with placenta previa or accreta. They modified and simplified Cho's method, requiring only one stitch instead of four. Parallel vertical compression sutures avoided the construction of a square dead space, which would prevent complications such as pyometra (19). Dedes et al. (20) achieved satisfactory results in six cases with the use of circular isthmic-cervical sutures to control peripartum hemorrhage in placenta previa accreta. However, this method interrupted blood circulation of the uterine artery, which could affect the recovery of uterine blood circulation. In recent studies, Li et al. (12) reported the use of funnel compression sutures to control postpartum bleeding during CS in the presence of placenta previa with or without placenta accrete; Ratiu et al. (21) performed parallel vertical compression sutures, but the reduction in postpartum hemorrhage was not as advantageous as traditional surgical methods in cases of placenta increta and placenta percreta.

Our modified procedure, parallel loop binding compression suture, can be applied in all types of placental abnormalities to reduce lower uterine segment bleeding. Using our method in patients with placenta increta or percreta, the average blood loss was 2,152.6 ± 1,169.4 mL, which was lower than control group (2,960.5 ± 1,963.6), and the infusion of blood products was significantly reduced. Although we selected patients with pernicious placenta previa complicated with placenta increta, however, our method was still effective at controlling lower uterine segment bleeding without hysterectomy compared to a previously reported method by Li (12), [92.11% (35/38) vs. 86.7% (18/22, respectively)].

Our modified method is similar to BH Zhao's transverse parallel compression suture (22). The main differences between the two methods are as follows: First, as an authoritative hospital in China, we usually accept a large number of patients with critical conditions and poor health status. At the same time, the inclusion criteria for our study are more stringent and we enrolled patients who were diagnosed with pernicious placenta previa with placenta increta; therefore, our study provides guidance for complex and difficult situations. Second, BH Zhao's study routinely preset the balloon catheter occlusion of bilateral iliac artery in all patients on the day of surgery. However, the rate of preoperative abdominal aortic balloon placement in our study was 26.32% (10/38). A randomized controlled trial reported that hospitalization costs and the rate of postoperative fever with internal iliac artery balloon occlusion were higher than others; further, balloon catheter occlusion of bilateral iliac artery did not reduce the volume of blood transfusion in patients with placental abnormalities and might increase unexpected bleeding risk (23). Third, BH Zhao's study started suturing 1–2 cm below the cesarean incision. In our method, we start stitching from the upper edge of the cervix and have 3–4 stitches to completely seal the bleeding on the placental dissection surface of the upper cervix and lower uterus. Our bottom-up suture method is conducive to fully exposing the bleeding site and avoiding the construction of a dead space in order to achieve more effective hemostasis.

The mean fetal childbirth time of 14.1 ± 5.6 min was longer than that of the control group (11.0 ± 8 min), but it was better for bladder protection and did not increase the rate of hospitalization in the NICU. The duration of indwelling catheter was significantly shortened in the study group, which is of great significance to the postoperative recovery of patients. All neonatal complications have been reasonably treated and eventually cured. Therefore, such surgical method and anesthesia time do not affect the outcomes of neonates. Parallel loop binding compression suture procedure is worthy of choice.

Unfortunately, some patients missed the optimal time to terminate the pregnancy due to lack of regular prenatal examination in our hospital. Three patients in study group underwent hysterectomy due to complete penetration of the placenta through the lower uterine segment. Based on ultrasound and MR evaluation, hysterectomy might be avoided if the pregnancy could be terminated earlier. Thus, planned termination of pregnancy needs to balance the risk of postpartum hemorrhage and the risk of prematurity. Placental abnormalities are associated with considerable maternal and fetal morbidity and even mortality. Maternal complications are mainly the result of massive hemorrhage (10). Therefore, it is essential to regular check-ups and timely termination of pregnancy.

The counseling regarding the subsequent pregnancy is also important. It should be emphasized that subsequent pregnancy is not recommended and effective contraception is necessary. As the number of cesarean sections increases, the probability of placental abnormalities is significantly higher, especially in patients with a history of placenta previa (24), which again puts the patient at risk of postpartum bleeding, hysterectomy and even death. Studies have shown that the hysterectomy rate was 6% in primary cesarean delivery for placenta previa, however, the rate was 25% for patients with placenta previa undergoing repeat cesarean delivery (25). For women with a strong desire for pregnancy, it is recommended to follow the WHO guidelines for contraception for at least 2 years (26), but it is necessary to perform a strict ultrasound examination to pay attention to whether there is a cesarean scar pregnancy and recurrent placental abnormality. In addition, assisted reproductive technology will also significantly increase the risk of placenta previa, which should be noted (27).

There are several highlights in our parallel loop binding compression suture. (1) We chose 35–38 weeks as a routine termination time of pregnancy. There were less risks than those chose delivery before 34 weeks (28). Among 38 patients with our method, 27 (71.05%) patients chose to terminate the pregnancy at 35–38 weeks. Others depended on the patient's clinical symptoms and evaluation based on ultrasound and MRI. (2) Our method is conducive to fully exposing the bleeding site and can extend the area of hemostasis to the whole lower uterus. (3) It is simple to operate, and conducive to rapid hemostasis. (4) Parallel loop binding compression suture is also effective in preventing hysterectomy. (5) Adjacent organs were not damaged.

## Conclusion

Parallel loop binding compression suture is an effective, swift, practical, and safe method to reduce postpartum bleeding in women with pernicious placenta previa, complicated with placenta increta. Besides, it has no adverse effects on newborns.

## Data Availability Statement

The original contributions presented in the study are included in the article/Supplementary Material, further inquiries can be directed to the corresponding authors.

## Ethics Statement

The studies involving human participants were reviewed and approved by Ethics Committee of Qilu Hospital. The patients/participants provided their written informed consent to participate in this study. Written informed consent was obtained from the individual(s) for the publication of any potentially identifiable images or data included in this article.

## Author Contributions

MF: data curation and writing—original draft preparation. HB: methodology, visualization, and writing—original draft preparation. YF, ML, and YX: data curation and supervision. CJ: methodology and software. XS, CW, LZ, and YZ: data curation. YX and LC: conceptualization and writing—reviewing and editing. All authors helped to perform the research and agreed to be accountable for all aspects of the work.

## Conflict of Interest

The authors declare that the research was conducted in the absence of any commercial or financial relationships that could be construed as a potential conflict of interest.

## Publisher's Note

All claims expressed in this article are solely those of the authors and do not necessarily represent those of their affiliated organizations, or those of the publisher, the editors and the reviewers. Any product that may be evaluated in this article, or claim that may be made by its manufacturer, is not guaranteed or endorsed by the publisher.

## Supplementary Material

Supplementary Video Clips 1–3 Parallel loop binding compression suture procedure.
