# Supplementary material for: Parallel Loop Binding Compression Suture, a Modified Procedure for Pernicious Placenta Previa Complicated With Placenta Increta
Source: Front Surg. 2021 Nov 29;8:786497. doi: 10.3389/fsurg.2021.786497 (PMC8666452; doi:10.3389/fsurg.2021.786497)
Supplement: Supplementary file 1 [file Table_1.DOCX]

## Supplementary material 1. Clinical characteristics of control group.

| **No.** | **Age** | **GA (week)** | **G/P/A** | **No. of CS** | **Fetal childbirth time(min)** | **Total time (min)** | **Blood loss (ml)** | **Blood Transfusion** | **Urethral catheter (days)** | **Antibiotic** | **Post-operation** |
| --- | --- | --- | --- | --- | --- | --- | --- | --- | --- | --- | --- |
|  |  |  |  |  |  |  |  | **(unit RBC/ ml FFP)** |  | **(days)** | **(days)** |
| 1 | 46 | 38+2 | 4/1/2 | 1 | 18 | 150 | 1200 | 6/400 | 2 | 2 | 2 |
| 2 | 38 | 36+4 | 4/2/1 | 1 | 5 | 81 | 1000 | 0/0 | 2 | 4 | 6 |
| 3 | 33 | 37 | 6/3/2 | 3 | 29 | 165 | 1300 | 0/0 | 2 | 3 | 5 |
| 4 ^a^ | 38 | 37+3 | 4/1/2 | 1 | 6 | 111 | 1500 | 6/400 | 3 | 4 | 4 |
| 5 ^a^ | 33 | 36+4 | 5/2/2 | 2 | 22 | 160 | 2000 | 6/600 | 6 | 5 | 6 |
| 6 ^a^ | 34 | 36+5 | 2/1/0 | 1 | 6 | 69 | 600 | 2/200 | 2 | 5 | 5 |
| 7 | 37 | 34+6 | 3/1/1 | 1 | 4 | 135 | 1200 | 0/0 | 2 | 3 | 8 |
| 8 | 37 | 37 | 6/4/1 | 3 | 14 | 120 | 1500 | 6/650 | 5 | 5 | 5 |
| 9 | 31 | 34+3 | 3/2/0 | 1 | 16 | 85 | 500 | 4/0 | 5 | 3 | 7 |
| 10 ^a^ | 35 | 38+3 | 5/1/3 | 1 | 7 | 168 | 2000 | 8/750 | 8 | 6 | 13 |
| 11 | 39 | 35+3 | 5/1/3 | 1 | 15 | 100 | 3500 | 10/600 | 5 | 5 | 6 |
| 12 | 38 | 37+1 | 4/3/0 | 3 | 9 | 160 | 2500 | 4/400 | 2 | 4 | 4 |
| 13 | 33 | 35 | 4/1/2 | 1 | 14 | 86 | 4500 | 10/1050 | 4 | 5 | 5 |
| 14 ^c^ | 29 | 37+1 | 3/1/1 | 1 | 5 | 170 | 4500 | 10/1000 | 6 | 6 | 6 |
| 15^b,d^ | 30 | 33+6 | 6/1/4 | 1 | 26 | 325 | 6500 | 40/2800 | 14 | 8 | 18 |
| 16 ^e^ | 37 | 35+1 | 4/2/1 | 2 | 5 | 170 | 5000 | 20/1600 | 2 | 7 | 8 |
| 17 | 37 | 36+1 | 3/1/1 | 1 | 6 | 98 | 3000 | 6/650 | 2 | 5 | 7 |
| 18 ^a^ | 43 | 36+1 | 4/1/2 | 1 | 5 | 120 | 2000 | 8/800 | 7 | 5 | 7 |
| 19 | 39 | 35+1 | 4/2/1 | 1 | 20 | 80 | 2000 | 4/400 | 3 | 5 | 6 |
| 20 ^f^ | 27 | 36+2 | 5/2/2 | 2 | 10 | 117 | 4000 | 12/1200 | 5 | 5 | 5 |
| 21 | 27 | 34+5 | 4/2/1 | 2 | 24 | 100 | 2000 | 8/800 | 3 | 3 | 5 |
| 22 | 32 | 35 | 5/2/2 | 2 | 27 | 110 | 2000 | 8/800 | 3 | 3 | 8 |
| 23 ^f^ | 37 | 36+4 | 6/1/4 | 1 | 8 | 68 | 4500 | 10/950 | 5 | 4 | 7 |
| 24 | 33 | 36+4 | 4/1/2 | 1 | 17 | 172 | 3500 | 14/1400 | 9 | 9 | 9 |
| 25 ^g^ | 31 | 37+1 | 2/1/0 | 1 | 7 | 105 | 3000 | 14/1400 | 2 | 3 | 8 |
| 26 | 38 | 37+1 | 6/2/3 | 2 | 26 | 130 | 2000 | 4/400 | 2 | 3 | 5 |
| 27 | 28 | 36+2 | 3/1/1 | 1 | 5 | 91 | 1200 | 2/0 | 2 | 3 | 5 |
| 28 ^a^ | 30 | 36+4 | 5/1/3 | 1 | 6 | 120 | 2000 | 8/500 | 2 | 3 | 4 |
| 29 | 32 | 36+5 | 2/1/0 | 1 | 4 | 85 | 3000 | 12/1200 | 9 | 5 | 10 |
| 30 ^a^ | 35 | 37 | 3/1/1 | 1 | 6 | 150 | 1800 | 4/400 | 2 | 4 | 5 |
| 31 | 40 | 39+2 | 4/1/2 | 1 | 30 | 125 | 2000 | 4/400 | 3 | 5 | 5 |
| 32 | 39 | 37+4 | 2/1/0 | 1 | 12 | 170 | 3000 | 10/100 | 4 | 5 | 5 |
| 33 | 41 | 35+6 | 4/2/1 | 2 | 4 | 193 | 10000 | 40/3200 | 5 | 6 | 7 |
| 34 | 36 | 39 | 3/1/1 | 1 | 7 | 81 | 1000 | 4/400 | 2 | 3 | 3 |
| 35 ^b^ | 29 | 35+5 | 2/1/0 | 1 | 3 | 208 | 8000 | 26/2600 | 13 | 7 | 14 |
| 36 | 31 | 35 | 4/1/2 | 1 | 20 | 100 | 2500 | 8/600 | 3 | 3 | 4 |
| 37 | 35 | 37+2 | 5/2/2 | 2 | 7 | 120 | 3000 | 8/800 | 5 | 6 | 6 |
| 38 ^h^ | 40 | 36+4 | 5/2/2 | 2 | 5 | 165 | 6000 | 18/2000 | 7 | 7 | 13 |
| 39 | 36 | 35+1 | 2/1/0 | 1 | 11 | 300 | 7500 | 26/2200 | 14 | 6 | 16 |
| 40 | 32 | 36+4 | 4/1/2 | 1 | 10 | 95 | 2000 | 6/500 | 2 | 3 | 6 |
| 41 | 31 | 37+6 | 4/1/2 | 1 | 7 | 75 | 2000 | 4/400 | 2 | 3 | 4 |
| 42 | 34 | 34+4 | 4/1/2 | 1 | 5 | 75 | 2000 | 4/400 | 1 | 4 | 5 |
| 43 | 26 | 37+2 | 4/1/2 | 1 | 25 | 115 | 2000 | 6/400 | 6 | 5 | 6 |
| 44 | 35 | 36+3 | 3/1/1 | 1 | 6 | 155 | 2000 | 6/700 | 3 | 3 | 7 |
| 45 ^i^ | 30 | 37 | 4/1/2 | 1 | 41 | 169 | 3000 | 8/800 | 5 | 4 | 6 |
| 46 | 32 | 38+1 | 3/1/1 | 1 | 15 | 120 | 1000 | 4/0 | 3 | 4 | 4 |
| 47 | 27 | 36+3 | 2/1/0 | 1 | 15 | 90 | 2000 | 4/400 | 1 | 3 | 5 |
| 48 | 30 | 36+4 | 3/1/1 | 1 | 3 | 88 | 2000 | 6/400 | 2 | 4 | 5 |
| 49 | 26 | 33+2 | 2/1/0 | 1 | 5 | 220 | 4000 | 14/1500 | 3 | 4 | 5 |
| 50 | 33 | 35+4 | 4/1/2 | 1 | 11 | 70 | 1000 | 6/800 | 2 | 4 | 5 |
| 51 | 33 | 36+1 | 3/1/1 | 1 | 3 | 85 | 3000 | 12/1200 | 3 | 7 | 7 |
| 52 ^h^ | 33 | 31+6 | 5/1/3 | 1 | 25 | 150 | 5000 | 20/1750 | 4 | 4 | 6 |
| 53 | 39 | 35+6 | 5/1/3 | 1 | 3 | 90 | 1500 | 10/1200 | 3 | 6 | 6 |
| 54 | 30 | 36+5 | 3/1/1 | 2 | 4 | 88 | 2000 | 6/600 | 4 | 5 | 5 |
| 55 ^h^ | 30 | 36 | 3/1/1 | 1 | 5 | 165 | 4000 | 10/1400 | 6 | 5 | 7 |
| 56 ^h^ | 29 | 39+1 | 3/1/1 | 1 | 5 | 81 | 3000 | 10/1800 | 2 | 5 | 6 |
| 57 ^h^ | 32 | 37+1 | 4/1/2 | 1 | 15 | 130 | 4500 | 18/1800 | 3 | 5 | 5 |
| 58 | 32 | 37 | 5/2/2 | 2 | 15 | 102 | 3500 | 12/1200 | 2 | 4 | 4 |
| 59 ^h^ | 36 | 35+2 | 3/1/1 | 1 | 5 | 115 | 5000 | 14/1900 | 3 | 4 | 5 |
| 60 | 35 | 36+3 | 4/1/2 | 1 | 7 | 145 | 1500 | 6/500 | 3 | 5 | 5 |
| 61^h,j^ | 37 | 33+3 | 3/1/1 | 1 | 5 | 282 | 5000 | 28/2800 | 6 | 6 | 10 |
| 62^b,c^ | 31 | 37+1 | 5/1/3 | 1 | 13 | 167 | 2500 | 10/800 | 7 | 5 | 10 |
| 63^b,f^ | 35 | 39 | 4/2/1 | 2 | 5 | 170 | 4000 | 16/1600 | 7 | 6 | 8 |
| 64^b,d^ | 27 | 32 | 2/1/0 | 1 | 8 | 260 | 9000 | 44/4200 | 10 | 8 | 11 |
| 65^b,k^ | 26 | 35+1 | 2/1/0 | 1 | 4 | 273 | 8000 | 32/2450 | 2 | 5 | 9 |
| 66 ^h^ | 37 | 35+5 | 3/1/1 | 1 | 8 | 120 | 5000 | 20/1800 | 7 | 6 | 8 |
| 67 | 31 | 36 | 3/2/0 | 2 | 15 | 95 | 2100 | 8/800 | 3 | 4 | 7 |
| 68 ^a^ | 35 | 34+4 | 3/1/1 | 1 | 6 | 112 | 1700 | 2/200 | 2 | 4 | 5 |
| 69 | 38 | 35+3 | 5/2/2 | 2 | 9 | 130 | 1500 | 8/800 | 2 | 4 | 4 |
| 70 | 32 | 36+4 | 2/1/0 | 1 | 4 | 72 | 2000 | 6/600 | 7 | 4 | 8 |
| 71 | 28 | 34+4 | 2/1/0 | 1 | 6 | 89 | 2000 | 6/600 | 4 | 5 | 5 |
| 72 | 40 | 36+5 | 2/1/0 | 1 | 15 | 220 | 5500 | 20/2000 | 5 | 7 | 7 |
| 73 | 32 | 36+6 | 4/2/1 | 1 | 3 | 80 | 1000 | 4/200 | 1 | 2 | 4 |
| 74 | 22 | 35+5 | 5/2/2 | 1 | 7 | 75 | 3400 | 8/600 | 3 | 4 | 8 |
| 75 | 29 | 36+4 | 4/2/1 | 2 | 6 | 80 | 2500 | 6/0 | 4 | 4 | 7 |
| 76 ^a^ | 35 | 35+1 | 6/2/3 | 2 | 6 | 104 | 1000 | 2/250 | 2 | 6 | 9 |
| 77 ^a^ | 38 | 36+4 | 5/1/3 | 1 | 5 | 147 | 2600 | 8/800 | 3 | 5 | 5 |
| 78 | 42 | 38+2 | 4/3/0 | 3 | 31 | 192 | 5000 | 16/1600 | 3 | 5 | 5 |
| 79 ^a^ | 34 | 33+5 | 5/2/2 | 2 | 8 | 146 | 1600 | 4/350 | 4 | 5 | 6 |
| 80 ^a^ | 43 | 37+3 | 2/1/0 | 1 | 14 | 120 | 800 | 2/200 | 4 | 5 | 5 |
| 81 ^a^ | 34 | 36+3 | 5/3/1 | 3 | 15 | 160 | 800 | 4/400 | 2 | 3 | 3 |
| 82 | 32 | 38+5 | 4/2/1 | 2 | 8 | 160 | 3500 | 16/1400 | 7 | 7 | 7 |
| 83 ^a^ | 38 | 36+5 | 6/2/3 | 2 | 13 | 115 | 800 | 4/400 | 2 | 3 | 5 |
| 84 | 36 | 34+3 | 6/2/3 | 2 | 15 | 177 | 1500 | 10/1000 | 3 | 4 | 5 |
| 85 ^a^ | 41 | 37 | 2/1/0 | 1 | 9 | 165 | 5000 | 16/1600 | 4 | 4 | 4 |
| 86 | 41 | 38 | 4/2/1 | 2 | 8 | 165 | 2500 | 12/1600 | 3 | 4 | 4 |

## ^a^ Abdominal aortic balloon was placed preoperatively. ^b^ Hysterectomy was performed due to massive blood loss.

## ^c^ 16 units of cryoprecipitate were infused. ^d^ 2 units of platelet were infused. ^e^ 12 units of cryoprecipitate were infused.

## ^f^ 20 units of cryoprecipitate were infused. ^g^ 10 units of cryoprecipitate were infused. ^h^ 1 unit of platelet were infused.

## ^i^ 8 units of cryoprecipitate were infused. ^j^ 72 units of cryoprecipitate were infused. ^k^ 20 units of cryoprecipitate were infused.

G/P/A, Gravidity/Parity/Abortion; CS, Cesarean section; RBC, Red blood cells; FFP, Fresh frozen plasma.

**Supplementary material 2. Neonatal characteristics of control group**

| **No.** | **Neonatal birth weight(g)** | **Apgar scores**  **(1 min/5min)** | **Neonatal complication** | **Hospitalization days in NICU** | **Prognosis** |
| --- | --- | --- | --- | --- | --- |
| 1 | 3300 | 10/10 | None | 0 | Cure |
| 2 | 2440 | 8/10 | 2,3,4,7,8 | 8 | Cure |
| 3 | 3000 | 10/10 | None | 0 | Cure |
| 4 | 2950 | 9/10 | None | 0 | Cure |
| 5 | 3300 | 8/10 | 2,3,5,9 | 18 | Cure |
| 6 | 3000 | 10/10 | None | 0 | Cure |
| 7 | 2650 | 8/9 | 3 | 10 | Cure |
| 8 | 3400 | 10/10 | None | 0 | Cure |
| 9 | 2500 | 10/10 | None | 0 | Cure |
| 10 | 3100 | 10/10 | None | 0 | Cure |
| 11 | 2800 | 10/10 | None | 0 | Cure |
| 12 | 3650 | 10/10 | None | 0 | Cure |
| 13 | 3000 | 7/9 | 2,3,6,10 | 9 | Condition improved |
| 14 | 3150 | 10/10 | None | 0 | Cure |
| 15 | 1900 | 10/10 | 2,3 | 13 | Cure |
| 16 | 3200 | 10/10 | 2,11 | 7 | Cure |
| 17 | 3000 | 10/10 | None | 0 | Cure |
| 18 | 2700 | 10/10 | 2 | 8 | Condition improved |
| 19 | 2500 | 6/10 | 2,3,4 | 11 | Condition improved |
| 20 | 2500 | 10/10 | None | 0 | Cure |
| 21 | 2640 | 10/10 | 3,12 | unknown | unknow |
| 22 | 2890 | 10/10 | None | 0 | Cure |
| 23 | 2900 | 10/10 | None | 0 | Cure |
| 24 | 2300 | 4/9 | 6,13 | 8 | Condition improved |
| 25 | 3000 | 10/10 | None | 0 | Cure |
| 26 | 3200 | 10/10 | None | 0 | Cure |
| 27 | 2850 | 10/10 | None | 0 | Cure |
| 28 | 3800 | 10/10 | None | 0 | Cure |
| 29 | 3500 | 10/10 | None | 0 | Cure |
| 30 | 2800 | 10/10 | None | 0 | Cure |
| 31 | 3850 | 10/10 | None | 0 | Cure |
| 32 | 2900 | 7/8 | 1,3,4,6 | 10 | Condition improved |
| 33 | 3000 | 10/10 | 2 | 5 | Condition improved |
| 34 | 3000 | 10/10 | None | 0 | Cure |
| 35 | 2100 | 10/10 | None | 10 | Cure |
| 36 | 2685 | 5/6 | 3,6 | 12 | Cure |
| 37 | 3000 | 10/10 | None | 0 | Cure |
| 38 | 3750 | 10/10 | None | 0 | Cure |
| 39 | 3550 | 10/10 | None | 0 | Cure |
| 40 | 3000 | 10/10 | None | 0 | Cure |
| 41 | 3400 | 10/10 | None | 0 | Cure |
| 42 | 2550 | 10/10 | 2 | 10 | Condition improved |
| 43 | 3350 | 9/10 | None | 0 | Cure |
| 44 | 2400 | 10/10 | None | 0 | Cure |
| 45 | 2800 | 10/10 | None | 0 | Cure |
| 46 | 4150 | 10/10 | None | 0 | Cure |
| 47 | 3100 | 10/10 | None | 0 | Cure |
| 48 | 3350 | 10/10 | 2,3,7,15 | 7 | Cure |
| 49 | 2400 | 8/10 | 5 | 9 | Cure |
| 50 | 2950 | 9/10 | 3,4 | 13 | Cure |
| 51 | 3000 | 9/10 | None | 0 | Cure |
| 52 | 2350 | 10/10 | Unknown | Unknown | Unknown |
| 53 | 2600 | 10/10 | None | 0 | Cure |
| 54 | 3200 | 10/10 | None | 0 | Cure |
| 55 | 2300 | 10/10 | None | 0 | Cure |
| 56 | 3050 | 10/10 | None | 0 | Cure |
| 57 | 3450 | 10/10 | None | 0 | Cure |
| 58 | 3100 | 10/10 | None | 0 | Cure |
| 59 | 2700 | 10/10 | None | 0 | Cure |
| 60 | 2840 | 10/10 | None | 0 | Cure |
| 61 | 2100 | 9/9 | 3,13 | 9 | Cure |
| 62 | 3350 | 10/10 | None | 0 | Cure |
| 63 | 2900 | 10/10 | None | 0 | Cure |
| 64 | 2000 | 8/8 | 3,5,6 | 4 | Dead |
| 65 | 2300 | 6/10 | 2,3,6,10 | 8 | Condition improved |
| 66 | 3650 | 9/10 | 2,9,16 | 27 | Cure |
| 67 | 2800 | 8/9 | 2,3,5,7,15 | 16 | Condition improved |
| 68 | 2750 | 9/10 | None | 0 | Cure |
| 69 | 2600 | 9/10 | None | 0 | Cure |
| 70 | 2450 | 10/10 | None | 0 | Cure |
| 71 | 2500 | 10/10 | 3,10,14 | 11 | Condition improved |
| 72 | 3200 | 8/9 | Unknown | Unknown | Unknown |
| 73 | 3100 | 10/10 | None | 0 | Cure |
| 74 | 2900 | 10/10 | None | 0 | Cure |
| 75 | 3000 | 10/10 | None | 0 | Cure |
| 76 | 2900 | 8/9 | 2,3,5,17 | 17 | Cure |
| 77 | 2900 | 9/10 | None | 0 | Cure |
| 78 | 4550 | 10/10 | None | 0 | Cure |
| 79 | 2450 | 5/7 | 2,5,6,9,18 | 18 | Cure |
| 80 | 2900 | 10/10 | None | 0 | Cure |
| 81 | 2510 | 10/10 | None | 0 | Cure |
| 82 | 3300 | 8/9 | None | 3 | Cure |
| 83 | 3400 | 10/10 | None | 0 | Cure |
| 84 | 2650 | 7/9 | 2,3,6,17 | 21 | Cure |
| 85 | 3500 | 10/10 | 3,7 | 9 | Condition improved |
| 86 | 3100 | 10/10 | None | 0 | Cure |

Neonatal complication: 1=Neonatal hypoxic-ischemic encephalopathy; 2=Neonatal hyperbilirubinemia; 3=Neonatal pneumonia; 4=Neonatal anemia; 5=Neonatal respiratory distress syndrome; 6=Neonatal apnea; 7=Neonatal myocardial injury; 8=Neonatal hypothermia; 9= Neonatal sepsis; 10=Neonatal encephalopathy; 11=Neonatal hypoglycemia; 12=Neonatal jaundice; 13=Neonatal swallowing syndrome; 14=Hemolytic disease of newborn; 15=Neonatal pneumothorax; 16=Purulent meningitis of newborn; 17=Neonatal feeding intolerance; 18=Neonatal shock.
